# Impact of post-thaw incubation time on viability and neovascularization of cryopreserved human ovarian tissue

**DOI:** 10.1186/s13048-026-02328-y

**Published:** 2026-11-09

**Authors:** Alexandra Waldhauer, Laura Rafensteiner, Sabine Eberhart, Josef Lehner, Katharina Hancke, Karin Bundschu

**Affiliations:** Department of Gynaecology and Obstetrics, University Hospital Ulm, Prittwitzstr. 43, 89075 Ulm, Germany

**Keywords:** Fertility preservation, Ovarian tissue cryopreservation (OTC), Chorioallantoic membrane assay (CAM), Neovascularization, Transplantation

## Abstract

**Background:**

This study aimed to investigate the effects of delayed transplantation after thawing on the quality of cryopreserved human ovarian cortex tissue.

**Methods:**

Cryopreserved human ovarian cortex tissue samples from five oncological patients (mean age 26 ± 3.6 years; AMH > 3 µg/L; mean: 7.64 ± 2.24 µg/L; oncological entities: brain tumor, breast cancer (*n* = 2), Hodgkin lymphoma, non-Hodgkin lymphoma) undergoing ovarian tissue cryopreservation were analyzed immediately after thawing, following different culture periods (1 h, 2 h, 4 h, 24 h) at room temperature, and after grafting onto the chorioallantoic membrane (CAM) of fertilized chicken eggs for short-term culture. Follicle viability and relative differences in viable follicle counts were assessed by Neutral Red staining. Tissue morphology, proliferation, apoptosis, angiogenesis and molecular expression of selected ovarian tissue markers were evaluated by histology (hematoxylin and eosin), immunohistochemistry (proliferation: Ki-67, apoptosis: cleaved Caspase-3), blood vessel quantification on CAM, and quantitative polymerase chain reactions (RT-qPCRs; apoptosis: BCL-2, BAX, hypoxia: HIF1A, angiogenesis: VEGFA, proliferation: mTOR, YAP, and follicle activation: GDF9, BMP15). Culture medium quality was examined by pH and microbiological analyses.

**Results:**

Ovarian cortex samples incubated for less than 4 h after thawing displayed preserved tissue integrity and viability, with minimal follicle activation and stable medium quality. Samples subjected to delays of more than 4 h showed structural damage and reduced tissue viability as well as relative difference in viable follicle counts of up to 67% after 24 h and markedly impaired neovascularization with a mean blood vessel count difference of 52.35%.

**Conclusions:**

Human ovarian cortex tissue maintained viability and structural integrity when transplantation was performed within 4 h after thawing. Delays exceeding 4 h were associated with reduced neovascularization, impaired tissue integrity, and increased relative difference in viable follicle counts. The data support minimizing avoidable delay and using four hours only as a provisional handling target requiring confirmation.

**Trial registration:**

Not applicable.

**Clinical trial number:**

Not applicable.

**Supplementary Information:**

The online version contains supplementary material available at https://doi.org/10.1186/s13048-026-02328-y.

## Introduction

The clinical relevance of fertility preservation for oncological patients has increased substantially in recent years. This is attributed to increased chances of recovery for many oncological entities, as well as desire for improved long-term quality of life [1], including family planning and the opportunity to conceive. Nevertheless, effective forms of oncological therapy like radiation and gonadotoxic chemotherapy may cause impaired function or complete ovarian failure and consequently infertility [2].

Current established methods of fertility preservation include surgical transposition of ovaries, cryopreservation of oocytes and embryos following hormonal stimulation or ovarian tissue cryopreservation (OTC), and pharmacological ovarian suppression with GnRHa (Gonadotropin Releasing Hormone agonist) as an additional, albeit controversially discussed, approach [3–5].

Chemotherapy approaches benefit from an early treatment start, resulting in time-sensitive patient management and consequently restricting chances of hormonal stimulation for oocyte harvesting. Additionally, OTC is the only suitable method of fertility preservation for prepubertal girls [6, 7].

Currently, OTC consists of surgically removing ovarian cortex tissue followed by freezing, storage, thawing and subsequent transplantation. At present, more than 200 live births have been reported with a live-birth-rate of 25–33% [8–10]. Typically, transplantation occurs immediately after the thawing process [11]. However, timing of transplantation may be compromised by external factors, including sudden deterioration in patient health or prolonged occupied operating theater. Furthermore, thawing and rehydration require specialized equipment and trained staff, which may not be readily available at all clinics. Transporting thawed tissue to patients treated elsewhere could enable specialized centers to perform the required laboratory procedures, providing adequate tissue quality and follicle viability after transport. Currently, limited research has addressed the optimal interval between thawing and transplantation. Therefore, this study aimed to examine whether delaying transplantation for up to 24 h after thawing compromises follicle viability, tissue integrity, and neovascularization of cryopreserved human ovarian cortex.

## Methods

### Animal research

Fertilized chicken eggs used for the chorioallantoic membrane (CAM) model were cultured for a maximum of 12 days post-fertilization, before completion of neural tube formation and development of a functional nervous system. In accordance with local regulatory guidelines, experiments at this developmental stage were exempt from formal ethical review and approval.

### Ethical approval

This study was approved by the Ethics Committee of University Hospital Ulm (Approval No. 181/17). The use of ovarian tissue for patient-related research was authorized.

### Study setup

**Fig. 1:**
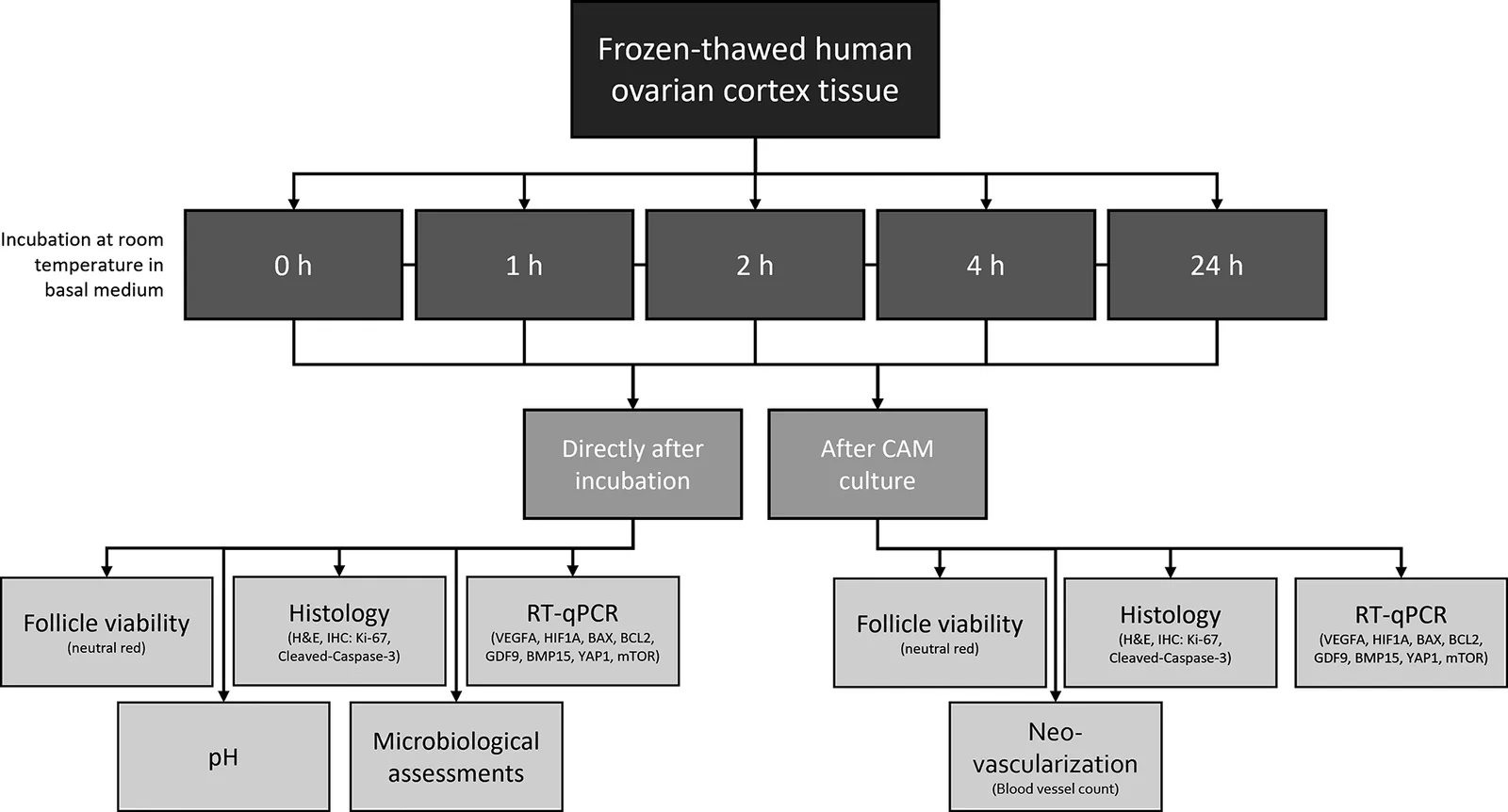
Experimental study design. Frozen-thawed human ovarian cortex samples were divided into five groups (0 h, *n* = 47; 1 h, *n* = 50; 2 h, *n* = 47; 4 h, *n* = 48; 24 h, *n* = 49). Samples were assessed directly after the assigned incubation interval or after a four-day CAM assay. Medium quality was evaluated by analyzing pH levels and microbiological assessments. Follicle viability was examined by Neutral Red stainings. Tissue integrity was analyzed histologically (H&E) and immunohistochemically (proliferation: Ki-67, apoptosis: cleaved Caspase-3). Gene expression analysis (RT-qPCRs) included markers for apoptosis (BCL-2, BAX), hypoxia (HIF1A), angiogenesis (VEGFA), proliferation (mTOR, YAP) and follicle activation (GDF9 and BMP15). Neovascularization was examined using the CAM model

Tissue fragments from each patient were randomly assigned to five groups, each subjected to a different post thaw storage duration (in hours): immediately (0 h), 1 h, 2 h, 4 h, and 24 h. Each tissue fragment (3 × 3 × 2 mm) was incubated in 6 mL basal medium (1% human serum albumin (PAN-Biotech, Aidenbach, Germany)) i​n Leibovitz L-15 medium (Gibco, Thermo Fisher Scientific, Germany) at room temperature in closed dishes without agitation for the respective storage interval before further processing (Fig. 1).

### Human ovarian tissue

Cryopreserved ovarian cortex tissue samples from five female patients (mean age: 26 ± 3.6 years) with different oncological entities (brain tumor, breast cancer (*n* = 2), Hodgkin lymphoma, non-Hodgkin lymphoma) and anti-mullerian hormone (AMH) levels above 3 µg/L (mean: 7.64 ± 2.24 µg/L) were analyzed. Tissue samples had previously been cryopreserved for fertility preservation using a slow freezing protocol according to Isachenko et al. [12]. Written informed consent for research use was obtained.

### Tissue processing

Frozen tissue samples were thawed under sterile conditions using the standard clinical thawing protocol. Briefly, samples were kept at RT (room temperature) for 2 min, followed by 2 min in a water bath at 37 °C. Subsequently, samples were washed in OvarStore thaw medium (basal medium with 1% human serum albumin (PAN-Biotech, Aidenbach, Germany) in Leibovitz L-15 medium (Gibco, Thermo Fisher Scientific, Germany) with 0.5 M sucrose and 17 µM gentamicin sulfate) for 15 min at RT, followed by gradual removal of cryoprotectants by adding 8 mL basal medium every 5 min over 30 min. After 2 min washing in basal medium, tissue strips were transferred to a sterile 10 cm dish containing basal medium and cut into 3 × 3 × 2 mm fragments. Then, samples were randomly allocated to five groups and incubated in 6 mL basal medium at RT for 0, 1, 2, 4 and 24 h before further processing. In total, 241 ovarian cortex fragments were assigned by analytical pathway: 91 to Neutral Red staining, 100 to histopathology, and 50 to RT-qPCR. Within each pathway, approximately half of the fragments were assessed directly after the assigned incubation interval and the remaining fragments after the four-day CAM assay. Supplementary Table 1 reports the group-specific allocation. The numbers therefore refer to analytical specimens, not to a single set of paired fragments (Suppl. Table 1).

### CAM culture

The CAM model was used to culture the tissue grafts. Fertilized chicken eggs were supplied by “LSL Rhein-Main Gefluegelvermehrungsbetriebe GmbH & Co KG”. Incubation and CAM culture were performed as previously described [13, 14]. Briefly, fertilized chicken eggs were incubated at 37 °C for 8 days before ovarian tissue grafting, followed by 4 days of graft culture prior to graft retrieval and further processing. Five independent experimental sets with a total of 199 chicken eggs were conducted (Suppl. Table 1).

### Neovascularization

Neovascularization was quantified after 4 days of CAM culture by imaging grafts in situ on day 12 (Stereomicroscope: model 47.50.57, Zeiss, Oberkochen, Germany; camera: Canon, Tokyo, Japan). Following excision with adjacent CAM tissue, samples were transferred to PBS-filled well plates, rotated to expose previously obscured vessels, and re-imaged. Blood vessels supplying each graft were quantified manually using ImageJ (RRID:​ SCR_003070).

### Follicle viability and count

Evaluation of follicle viability and count was performed after incubation for defined time points at RT and after CAM culture. Neutral Red staining allows the differentiation of viable and non-viable follicles [15] as previously described [13]. Follicles were classified as viable when both oocytes and granulosa cells were positively Neutral Red-stained. Because separate fragments were assessed before and after CAM culture, results are described as relative differences in viable follicle counts and not as paired follicle loss.

### Histology and Immunohistochemistry

Tissue samples for histology and immunohistochemistry were fixed in 4% formaldehyde solution (Otto Fischar, Saarbrücken, Germany) overnight. Following fixation, samples were dehydrated, embedded in paraffin and cut into 3 μm sections (RM2255, Leica Biosystems, Nussloch, Germany). Sections were transferred onto superfrost slides and incubated at 50 °C overnight. To assess tissue integrity, sections were stained with hematoxylin and eosin (H&E) using an automated stainer (ST5020, Leica, Germany) and evaluated by brightfield microscopy (Keyence BZ-X810, Osaka, Japan). Tissue vitality was analyzed using immunohistochemistry with Ki-67 (cell proliferation) and cleaved Caspase-3 (apoptosis) as markers. Deparaffinization was performed using Neoclear (Merck), followed by rehydration, antigen retrieval with citrate buffer (pH 6.0), and blocking of endogenous peroxidase activity with 3% hydrogen peroxide in methanol. Non specific binding sites were blocked with goat serum (1:20). Primary antibodies (Anti-Ki-67, 1:600, Anti-cleaved Caspase-3, 1:100; Cell Signaling Technology, Cambridge, UK) were incubated overnight at 4 °C, followed by tris-buffered saline (TBS) washing and incubation for 30 min with biotinylated secondary antibodies (Ki-67: anti-mouse IgG, 1:200, cleaved Caspase-3: anti-rabbit IgG, 1:200; Invitrogen, Germany). Visualization was performed using a Streptavidin-coupled Peroxidase and DAB substrate kit (Thermo Fisher, Rockford, USA). Slides were mounted with Aquatex^®^ (Merck) and cover slips. Negative controls were performed in parallel lacking primary antibodies. The stained areas were quantified using a brightfield microscope (Keyence BZ-X810) and ImageJ 2 (version 2.16.0/1.54p; RRID: SCR_003070).

### Reverse transcription quantitative PCR (RT-qPCR)

RT-qPCRs were performed as described before [14]. The following gene expressions were investigated: Vascular Endothelial Growth Factor A (VEGFA) and Hypoxia-Inducible Factor 1 Alpha (HIF1A) to examine neovascularization and hypoxia; BCL2-Associated X Protein (BAX) and B-Cell Lymphoma 2 (BCL2) as apoptosis-related proteins; and mammalian Target Of Rapamycin (mTOR), Yes Associated Protein 1 (YAP1), Growth-Differentiation Factor 9 (GDF9) and Bone Morphogenic Protein 15 (BMP15) to assess follicular development and activation. Endogenous Glyceraldehyde 3-phosphate dehydrogenase (GAPDH) was used as a standard housekeeping gene expression marker.

### Medium quality

To assess medium quality across incubation times, pH and potential microbiological contamination were investigated, with pH levels being determined using indicator strips (Hydrion, pH 6.0–8.0, Micro Essential Laboratory, New York, USA). For microbiological assessment, 100 µL of basal medium was plated on brain-heart-infusion agar (VWR, 37 g BHI/L purified water) and incubated at 37 °C for 5 days. Control plates without medium and plates containing fresh medium were processed in parallel. After culture, plates were evaluated for visible fungal or bacterial growth (Suppl. Figure 1).

### Statistics

Analyses were restricted to descriptive statistics because tissue was obtained from five patients and multiple fragments were analyzed per patient. GraphPad Prism version 10.6.0 (RRID: SCR_002798) was used for data organization, graphing, and descriptive summaries. Continuous variables are presented as mean ± SEM.

## Results

### Stable basal medium quality during 24 h incubation

Medium quality was monitored throughout the incubation period by assessing samples at predetermined time points (0, 1, 2, 4, and 24 h) (Suppl. Figure 1). The basal medium maintained a stable pH of 6.4 across all groups and time points. No relevant bacterial or fungal contamination was detected in any sample throughout the study.

### Extended delays before transplantation are associated with increased relative difference in viable follicle counts and impaired tissue integrity

The number of viable follicles in cortex samples was evaluated immediately after thawing, following defined delay times (1, 2, 4, 24 h), and after CAM culture. In total, 91 samples were examined (Suppl. Table 1). All viable primordial, primary and secondary follicles were counted. Absolute numbers of viable follicles varied substantially both between patients and between fragments from the same patient (Fig. 2A). No consistent changes were observed across groups when comparing absolute counts before and after CAM culture. However, when comparing mean relative difference in viable follicle counts, groups with storage time below 4 h (0 h, 1 h, 2 h) showed less than 25% relative difference in viable follicle counts following culture. Longer culture times of 4 h and 24 h were both associated with substantially higher relative difference in viable follicle counts of 67–85% (Fig. 2B).

Overall, expression of follicle activation markers remained low across all groups. YAP1 expression as part of the Hippo pathway was slightly higher in the 24 h group but remained low overall (Fig. 2C). mTOR as part of the PI3K/AKT pathway showed no consistent trend (Fig. 2D). Expression of BMP15 and GDF9 as general markers for follicle activation was minimal to undetectable in nearly all samples (Fig. 2E, F).

**Fig. ​2:**
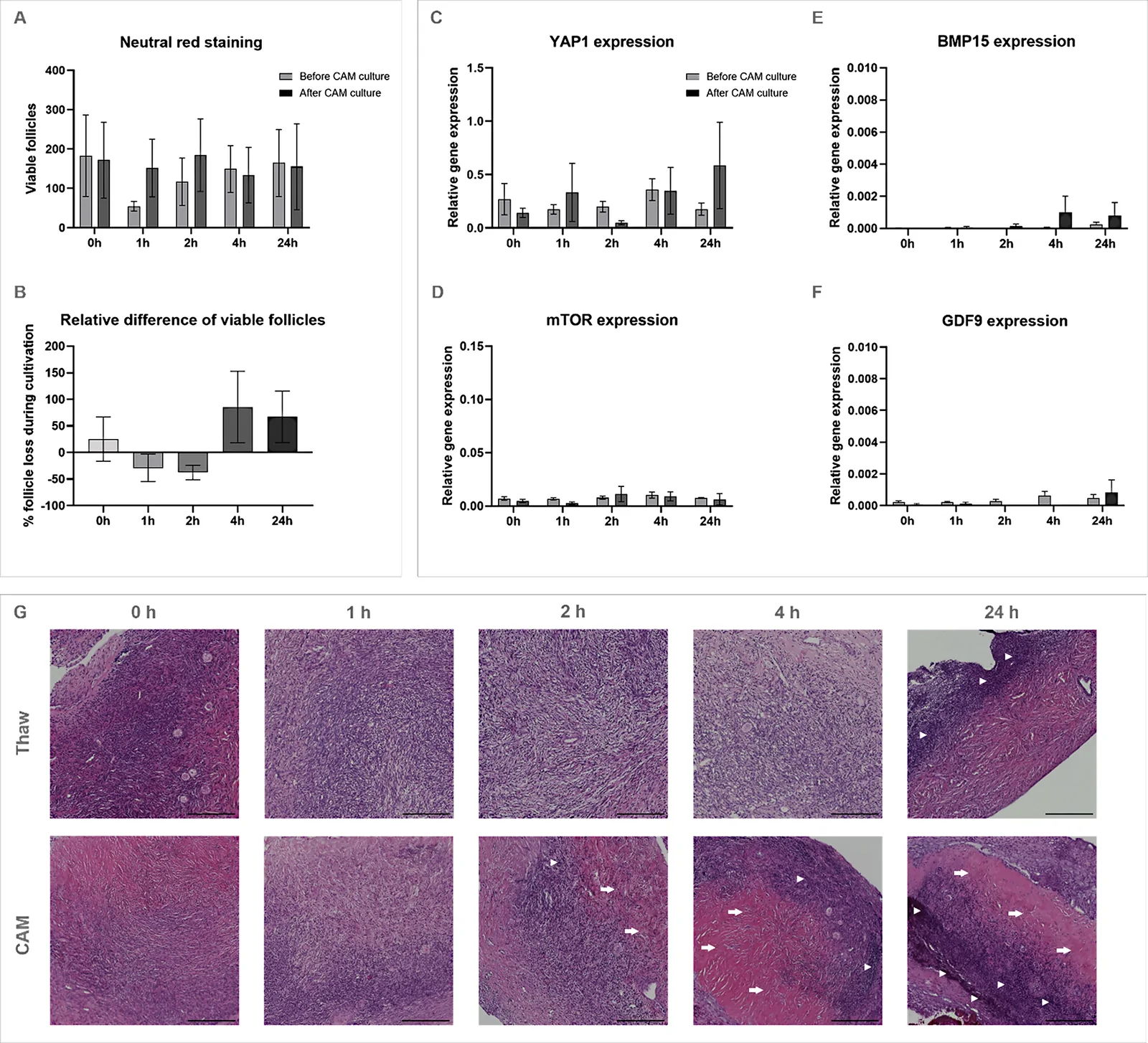
Follicle amount and activation. (**A**) Descriptive statistic of viable follicle count by mean ± SEM; (**B**) Descriptive statistic of relative difference in viable follicle counts by mean ± SEM; (**C**-**F**) Descriptive statistic of marker gene expression (follicle activation markers) in RT-qPCR analysis by mean relative gene expression ± SEM. (**G**) Tissue structure analysis with H&E staining. Representative pictures of H&E-stained tissue before and after CAM culture at different indicated time points. Increase in structurally impaired regions after longer delays before transplantation. Arrows indicate increased basophilic tissue regions. Arrow tips indicate cell-poor areas. Yes-associated Protein 1 (YAP1); mammalian Target of Rapamycin (mTOR); Bone Morphogenic Protein 15 (BMP15); Growth Differentiation Factor 9 (GDF9). Scale bars: 100 μm. (Thaw: directly after incubation; CAM: after CAM culture)

Before CAM culture, physiological ovarian tissue structure was observed across all observed time points (0–24 h), with rare areas of necrosis or internal tissue separation. However, after CAM culture, physiological tissue integrity appeared impaired, revealing an increase in heavily basophilic regions with cellular debris, and eosinophilic cell-poor areas (Fig. 2G). Regional demarcations and deformed nuclei in line with necrotic processes could be observed most prominently after 4 to 24 h of delay before transplantation. Nevertheless, physiological morphology and similar distribution of follicles were retained throughout culture in all test groups.

### Prolonged delay of transplantation was associated with increased apoptosis after CAM

**Fig. 3:**
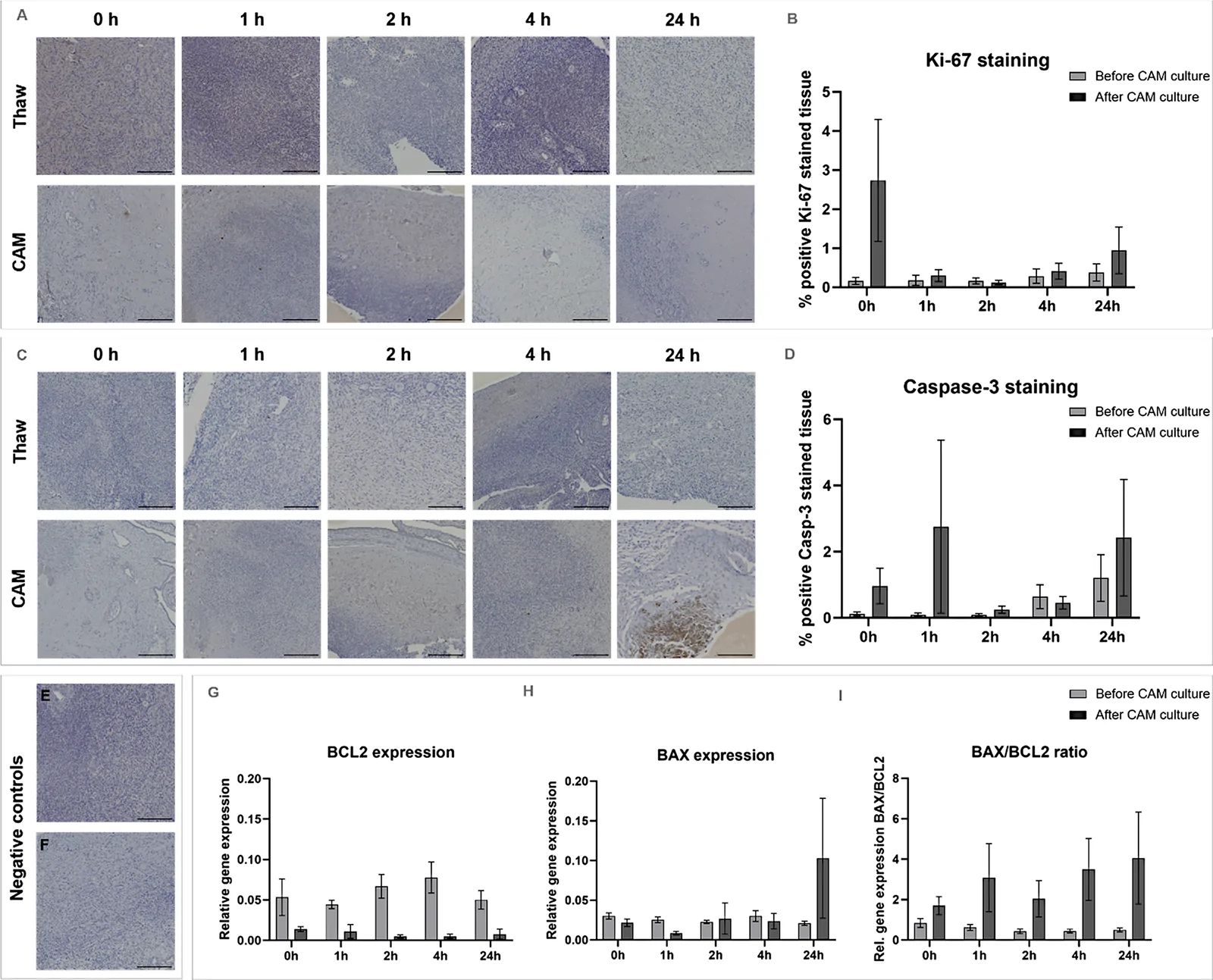
Tissue vitality. (**A**) Representative pictures of Ki-67-stained samples for each group (0 h; 1 h; 2 h; 4 h; 24 h) after indicated incubation times, before and after CAM culture; (**B**) Descriptive statistic of area % of Ki-67-stained tissue by mean ± SEM; (**C**) Representative pictures of cleaved Caspase-3-stained samples for each group (0 h; 1 h; 2 h; 4 h; 24 h) after indicated incubation times, before and after CAM culture; (**D**) Descriptive statistic of area % of cleaved Caspase-3-stained tissue by mean ± SEM; (**E**; **F**) Representative negative controls for (**E**) Ki-67 and (**F**) cleaved Caspase-3 lacking primary antibodies. (**G**-**I**) RT-qPCR analysis demonstrating mean relative marker gene expression ± SEM of apoptosis-related marker genes; (**G**) BCL2-Associated X protein (BAX); (**H**) B-Cell Lymphoma 2 protein (BCL2); (**I**) BAX/BCL2-ratio; Scale bars: 100 μm (**A**, **C**, **E**, **F**). (Thaw: directly after incubation; CAM: after CAM culture)

Proliferative and apoptotic activity remained low among all observed groups before transplantation onto CAM. After CAM culture, both proliferation (Ki-67-positive cell areas) and apoptosis (cleaved Caspase-3-positive cell areas) increased across all groups, most notably after 24 h delay. Furthermore, expression of apoptosis-marker BAX varied across groups, with a notable increase in the 24 h group after CAM culture. In contrast, the anti-apoptotic BCL2 expression marker decreased after culture across all groups. The ratio of pro-apoptotic to anti-apoptotic factors (BAX/BCL2-ratio) increased after CAM culture and reached the highest level in the 24 h group (Fig. 3).

### Reduced neovascularization and increased hypoxia after 4 h delay

**Fig. 4:**
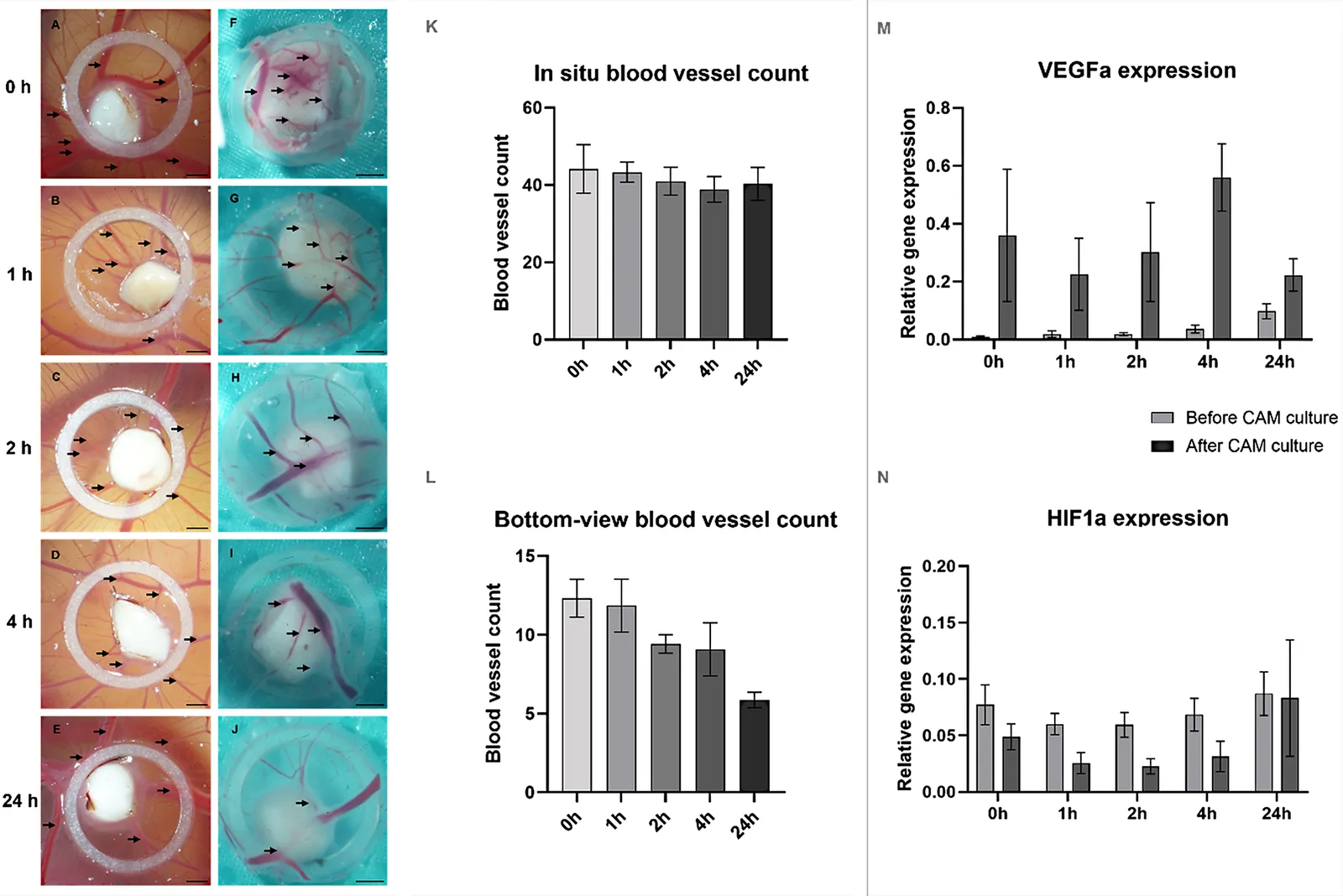
Reduced neovascularization and increased hypoxia after 24 h incubation followed by transplantation. (**A**-**E**) Representative pictures of CAM in situ with grafted tissue on day 12 of 4 days of CAM culture for each indicated group (0 h; 1 h; 2 h; 4 h; 24 h); (**F**-**J**) Representative pictures of removed tissue on day 12 from bottom view of the CAM; (**K**) Descriptive statistic of vessel count of the CAM in situ by mean ± SEM; (**L**) Descriptive statistic of directly transplant-supplying manual blood vessel count of the CAM from bottom side by mean ± SEM; (**M**; **N**) RT-qPCR analyses of angiogenesis-related gene expressions by mean relative gene expression ± SEM; (**M**​) Vascular Endothelial Growth Factor A (VEGFA); (**N**) Hypoxia-inducible Factor 1 alpha (HIF1A); Arrows exemplarily point to blood vessels; Scale bars: 1000 μm (**A**-**J**)

To assess neoangiogenesis and graft vascularization, blood vessels supplying the transplanted ovarian tissue samples were evaluated. Additionally, gene expression of angiogenesis markers VEGFA and HIF1A were examined. Although the number of vessels surrounding the grafts in situ on the CAM remained similar between groups, evaluation of vessels directly supplying the transplanted grafts revealed marked group-specific differences. In samples with 24 h delay before transplantation, the average number of vessels was considerably lower than in those of earlier groups. Compared to the control group transplanted directly after incubation (0 h) with an average of 12.32 ± 2.68 vessels, the 24 h group showed a 52.35% reduction with an average of 5.87 ± 1.09 directly supplying vessels. VEGFA expression before culture was low, with a slight increase observed after extended delay. After CAM culture, VEGFA expression strongly increased across all groups, with the 24 h group showing the lowest relative gene expression. HIF1A expression was generally low with a decrease after CAM culture. The lowest change and highest post-CAM culture expression was observed in the 24 h group (Fig. 4).

## Discussion

This study investigated whether delaying transplantation affects the short-term tissue integrity, follicle viability and neovascularization of cryopreserved human ovarian cortex. In this experimental model, prolonged post-thaw incubation, particularly for up to 24 h, was associated with impaired tissue architecture, increased apoptotic cell signaling, reduced neovascularization, and greater relative difference in viable follicle counts. However, tissue transplanted within 4 h after thawing showed largely preserved morphology and viability.

Although no evidence-based recommendations define the optimal interval between thawing and ovarian tissue transplantation, clinical practice generally favors minimizing this interval whenever feasible [11]. Unexpected delays, including patient-related complications immediately prior to transplantation or emergency surgical procedures necessitating short-term changes to operating theater schedules, may occur after ovarian tissue fragments have already been thawed and prepared in specialized laboratory facilities. This raises the question of how these grafts can be optimally handled and stored until transplantation. Our findings indicate that extending the post-thaw interval to up to 4 h does not appear to substantially compromise tissue viability or structural integrity. However, post-thaw intervals of 24 h were associated with impaired tissue architecture, reduced neovascularization, and increased relative difference in viable follicle counts.

### Preserved basal medium quality during storage

Basal medium was selected to mimic routine clinical storage conditions. pH values maintained stable throughout the whole incubation period, indicating preserved medium quality. Furthermore, microbiological analyses revealed no evidence of bacterial or fungal contamination originating from either the storage medium or the processing environment. These findings suggest that additional antibiotic supplementation of the basal medium is not required under the conditions investigated. Moreover, the rehydration protocol already incorporates OvarStore Thaw, which contains gentamicin, a broad-spectrum antibiotic. However, these findings apply only to the tested conditions and do not establish that antibiotic supplementation is unnecessary in clinical practice.

### Follicle viability

Physiologically, follicle loss is increasing with age, with a distinct reduction of both amount and density of viable dormant and developing follicles after the age of 35 [16]. Aside from age, AMH serves as a well-established marker for tissue composition and follicle reserve in clinical settings [17].

While the number of viable follicles varied markedly between patients (inter-individual) and between different samples from the same patient (intra-individual), relative difference in viable follicle counts showed a trend toward higher values if transplantation delays exceeding 4 h.

In groups with shorter storage intervals, relative difference in viable follicle counts did not exceed 25%, and in some cases, post-culture grafts even contained a higher number of viable follicles than corresponding pre-transplantation samples. This finding most likely reflects heterogeneously distributed follicles within the ovarian cortex and regional differences in follicular density, as previously described by Schenck et al. [18]. As the tissue samples were randomly allocated to the different experimental groups with downstream analyses following processing, variations in follicle counts were anticipated within individual patients. The increased relative difference in viable follicle counts observed after CAM culture in groups where transplantation took place more than four hours after thawing may reflect impaired neovascularization in conjunction with a progressive deterioration of the stroma.

### Minimal to no follicle activation

Following transplantation of frozen-thawed ovarian tissue, excessive activation and recruitment of quiescent primordial follicles may contribute to an accelerated follicular depletion [19]. To investigate possible differences between study groups, the expression of selected marker genes was analyzed by RT-qPCR. Both, the PI3K/AKT and Hippo signaling pathways regulate primordial follicle proliferation and cell differentiation [20–22]. mTOR and YAP1, the principal downstream effectors of PI3K/AKT and Hippo signaling pathways respectively, were selected for gene expression analysis. In addition, GDF9 and BMP15, both closely associated with follicular development, were investigated [23, 24].

Overall, gene expression levels were low across all examined genes and experimental groups, providing no evidence of increased follicle activation shortly after transplantation. Likewise, the expression of GDF9 and BMP15 ranged from very low to undetectable, with no consistent differences between groups. Expression of mTOR, a key downstream effector of the PI3K/AKT signaling pathway, was similarly low across all groups. Although YAP1 expression exhibited considerable inter-individual variability, overall expression levels remained low. Notably, Li et al. [25] interpreted expression levels of approximately 0.4 as negligible, whereas mean YAP1 expression in our samples was consistently lower. By comparison, biologically relevant expression levels reported in that study reached values of up to 1.5, considerably exceeding those observed in our samples. Collectively, these findings suggest minimal YAP1 activity in our experimental groups.

Similarly, low mTOR expression observed in our samples is consistent with previously reported baseline or minimally activated levels, further supporting the absence of marked follicular activation [26, 27]. These findings provide no evidence of relevant differences in follicular recruitment or activation between groups undergoing delayed transplantation and control groups.

### Tissue structure and vitality

Histological assessment of H&E-stained ovarian cortex tissue revealed increasing stromal damage and disruption of tissue architecture with prolonged post-thaw incubation. These alterations are likely attributable to cryopreservation-related stressors, including ice crystal formation and osmotic imbalance [19], and were most evident in the 24 h group.

Impaired stromal integrity may compromise the structural support required for neovascularization and long-term follicle survival. In agreement with the histological findings, immunohistochemical and molecular analyses indicated increased apoptotic activity in the 24 h post-thaw group, as reflected by the largest cleaved Caspase-3-positive area and the highest BAX/BCL2 ratio.

Ki-67 immunostaining revealed a generally low proliferative activity across all groups. As Ki-67 is a marker of cell proliferation, these findings are consistent with the assumption that recently thawed ovarian tissue has not yet regained its full proliferative potential [28]. Although a slight increase of Ki-67 positive areas was observed with longer post-thaw storage intervals, overall proliferative activity remained low.

### Reduced neovascularization and increased hypoxia after 4 h

Ischemic injury resulting from insufficient vascularization, together with premature follicle activation, represents one of the principal causes of ovarian graft failure following transplantation [19]. Macroscopic assessment of the CAM after 4 days of culture with the grafts in situ revealed negligible differences in the total number of visible vessels between the study groups. However, this approach did not allow reliable differentiation of vessels directly supplying the grafts. Following graft excision, assessment of the exposed CAM enabled a more accurate evaluation of the graft-specific vascular supply. Overall, hypoxia-related gene expression decreased after CAM culture, indicating successful re-establishment of oxygen supply. However, post-thaw incubation delays exceeding 4 h were associated with a marked reduction in the amount of vessels directly supplying the grafts. Compared with the 0 h control group, the total number of graft-feeding vessels was reduced by 52%. Consistent with these findings, the 4 h and 24 h groups exhibited the highest expression of hypoxia-associated genes following CAM culture. As expected, VEGFA expression increased after transplantation, reflecting the angiogenic response to graft implantation. However, VEGFA expression in the 24 h group was lower than in the other experimental groups, supporting impaired neovascularization at both the molecular and macroscopic levels. Given that ischemic injury is a major determinant of early graft failure [19], the observed reduction in graft vascularization may also explain the increased BAX/BCL2 ratio, impaired tissue architecture, and enhanced relative difference in viable follicle counts observed after prolonged post-thaw incubation.

### Clinical implications

The interval between thawing and transplantation represents a clinically important but insufficiently defined parameter in ovarian tissue cryopreservation (OTC) protocols. In our study, post-thaw intervals exceeding 4 h were associated with a trend toward impaired tissue integrity, reduced follicle viability, and compromised neovascularization.

Several studies have investigated the impact of the time interval between tissue retrieval and the start of cryopreservation on the viability of ovarian tissue. Their findings suggested that cryopreservation of ovarian tissue delayed by up to 24 h after collection did not impair the viability of the tissue, provided that samples were stored at 5 °C [12, 29]. Different storage temperatures during the interval between thawing and transplantation were not analyzed within this study, but may further impact tissue quality and could potentially extend the safe post-thaw storage period.

Although these findings are based on a small sample size and should therefore be interpreted with caution, they suggest that prolonged incubation following thawing may compromise the quality of the graft. From a clinical perspective, it therefore seems advisable to limit the time between thawing and transplantation to less than four hours until these results are confirmed in larger, controlled studies.

### Limitations and outlook

Although our data suggest that minimizing the interval before transplantation may be beneficial, larger studies including greater patient cohorts are required to validate these findings. Future investigations should further explore the impact of extended incubation periods and different storage temperatures, as the present study was limited to short-term CAM culture. Long-term post-transplantation assessment may be achieved using a murine transplantation model. Furthermore, Isachenko et al. demonstrated that pre-cooling of ovarian cortex tissue prior to cryopreservation may preserve follicular viability and mitigate cryoinjury [12]. Because the study included five patients with heterogeneous diagnoses, separate fragments rather than matched specimens, and descriptive analyses only, no validated clinical threshold can be inferred. Building on these findings, future studies should evaluate post-thaw storage conditions by comparing ovarian cortex samples maintained at room temperature with those stored under hypothermic conditions (4–5 °C). Larger patient-level studies, confirmed incubation details, blinded or automated vascular quantification, comparison of storage temperatures, and long-term mammalian transplantation models are required.

## Conclusion

This study demonstrates that prolonged intervals between thawing and transplantation exceeding 4 h negatively affect the quality of frozen-thawed human ovarian cortex tissue. Compared with shorter or no incubation intervals, prolonged delays were associated with greater disruption of tissue architecture and integrity, increased apoptosis, enhanced relative difference in viable follicle counts after CAM culture, and elevated hypoxic stress, due to impaired angiogenesis. In contrast, samples incubated for less than 4 h showed no substantial evidence of decline in tissue quality compared with controls. Our findings suggest that transplantation within four hours after thawing may represent a provisional handling target for cryopreserved human ovarian tissue. However, this timeframe should not be considered a validated clinical deadline, and further studies are required to confirm its relevance for tissue viability, neovascularization, and clinical outcomes.

## Supplementary Information

Below is the link to the electronic supplementary material.

Supplementary Material 1

## Data Availability

The datasets used and/or analyzed during the current study are available from the corresponding author on reasonable request.

## Declarations

### Research resource identifiers

ImageJ (RRID:​ SCR_003070); GraphPad Prism (RRID: SCR_002798).
