# Supplementary material for: Impact of post-thaw incubation time on viability and neovascularization of cryopreserved human ovarian tissue
Source: J Ovarian Res. 2026 Nov 9;19:341. doi: 10.1186/s13048-026-02328-y (PMC13757142; doi:10.1186/s13048-026-02328-y)
Supplement: Supplementary file 1 [file 13048_2026_2328_MOESM1_ESM.docx]

**Supplementary Material**

***Suppl. Figure 1:*** *Medium quality monitoring. (A) Representative picture of an agar plate containing BHI-agar and basal medium - all samples appeared macroscopically free from contaminations after 5 days incubation at 37° C. (B) Representative picture of pH analysis using pH indicator paper.*

***Suppl. Table 1:*** Sample distribution of ovarian cortex tissue fragments across study groups and corresponding methodology.

|  | **0h** | | **1h** | | **2h** | | **4h** | | **24h** | | **Sum** |
| --- | --- | --- | --- | --- | --- | --- | --- | --- | --- | --- | --- |
|  | **Thaw** | **CAM** | **Thaw** | **CAM** | **Thaw** | **CAM** | **Thaw** | **CAM** | **Thaw** | **CAM** |  |
| **Neutral Red Staining** | 9 | 8 | 10 | 10 | 8 | 9 | 10 | 8 | 10 | 9 | 91 |
| **Histopathology** | 10 | 10 | 10 | 10 | 10 | 10 | 10 | 10 | 10 | 10 | 100 |
| **RT-qPCR** | 5 | 5 | 5 | 5 | 5 | 5 | 5 | 5 | 5 | 5 | 50 |
| **Sum** | 24 | 23 | 25 | 25 | 23 | 24 | 25 | 23 | 25 | 24 | 241 |
| **Sum total** | 47 | | 50 | | 47 | | 48 | | 49 | | 241 |

***Suppl. Table 1:*** *Each graft was transplanted onto one fertilized chicken egg for culture. (Thaw: directly after incubation; CAM: after CAM culture).*
